# The role of physical activity in the physiological activation of the scholastic pre-requirements

**DOI:** 10.3934/Neuroscience.2024016

**Published:** 2024-08-19

**Authors:** Francesca Latino, Francesco Tafuri

**Affiliations:** 1 Department of Psychology and Education, Pegaso University, 80100 Naples, Italy; 2 Heracle Lab Research in Educational Neuroscience, Niccolò Cusano University, 00166 Rome, Italy

**Keywords:** physiology, academic performance, cognitive function, exercise, physiological factors

## Abstract

Physical activity during the developmental age is an indispensable tool for the physical and mental growth of children. Thanks to physical activity, individuals have the opportunity to improve their physical efficiency and promote better health, establish relationships with the environment and with others, and develop cognitive processes. Therefore, the aim of this study is to investigate the relationship between physical activity and the development of scholastic prerequisites among kindergarten children. 52 children (aged 4–5) participated in either a classroom-based physical activity program (60′/3 days per week) or regular lessons. At the beginning and end of the intervention programs, a set of standardized motor evaluation tests and the Observational Questionnaire for the Early Identification of Learning Disabilities (IPDA) were administered. As a result, a meaningful Time x Group interaction for the IPDA Variable was observed. The aforementioned development denotes a noteworthy advancement within the treatment group (p < 0.001). Conversely, no substantial modification was noted in the control group. The findings derived from this study provide a foundational support to the concept that physical activity integrated into classroom settings is an effective strategy to improve both scholastic prerequisites and academic performance.

## 1. Introduction

In recent years, the scientific community has recognized the body and movement not only as the same dignity as other intellectual forms, but a dimension thanks to which they are recognized as accelerators of human learning processes [1]. Starting from these assumptions, a growing scientific interest has developed in the effects of physical activity on cognitive functioning and brain health [2]. This interest has been largely driven by the contribution of neuroscientific studies that have linked physical activity to cognitive function, as well as to brain structure and function [3]. The first evidence of the direct effects of exercise on the brain was obtained from research conducted on animals [4]. Subsequently, a growing body of data from laboratory studies began to provide the first evidence about the potential cellular and molecular mechanisms through which physical activity was able to influence brain structure and function, with concomitant benefits on cognitive processes [5].

Cognitive processes are those functions through which the person can direct attention, acquire and process information from the surrounding world, recall the most important information and neglect the less important ones, and make decisions in relation to the needs [6]–[11]. Over the last few decades, numerous studies have shown that physical activity has a clear influence on cognitive functions, especially on processes which involve learning skills and memory [12]. It is able to improve the fundamental aspects that underlie school skills, such as concentration, working memory, classroom behavior, self-esteem, and a sense of perceived self-efficacy [13]. In fact, physical activity can positively regulate brain processes related to learning mechanisms, which induce significant functional and structural changes at the level of the Central Nervous System (CNS).

The relationship between motor development and cognitive performance was probably first established in children [14]; however, evidence of the benefits of exercise on human cognition has been more fully developed in research with older adults. Many of these experiments have clearly shown that regular physical activity alters specific brain structures and functions, which can lead to an improvement in cognitive performance [15]. Already in early academic papers, researchers concluded that children with better physical fitness showed a greater allocation of attentional resources to working memory [16]. In addition, Sibley and Etnier [17], who more than any other have laid the foundations of research in this field, have confirmed the existence of a small but significant connection between physical activity and cognitive performance in school-age children (4 years), and suggested that physical activity may be related to positive cognitive outcomes during development.

Early scientific studies on the relationship between physical activity and cognitive functions suggested that even short-term exercise was able to improve several aspects of cognitive function immediately after the completion of the activity, regardless of the level of physical efficiency [18]. However, until recently, the relationship between strenuous exercise and cognitive function was not fully established since the literature on the subject seemed to provide somewhat contradictory results. In fact, while several studies indicated that short sessions of physical activity could induce benefits on cognitive functioning [19], others found no beneficial effect [20].

In recent years, research in this field has considerably evolved through studies born from the need to better understand the multifaceted relationship between physical activity and cognitive health. Advances in neuroscience and neuroimaging techniques have allowed researchers to better understand the effects of physical activity on cognitive functions by investigating their physiological activation and molecular mechanisms [5]. The research conducted in the different areas of interest of physical activity has helped to define this line of investigation and to identify some underlying preliminary mechanisms. This has led to a deeper understanding of the components that are related to cognitive function and those that may be amenable to intervention [21]. In more recent times, it has been shown that the effect of physical activity on cognitive performance depends on both the intensity and duration of exercise [22], as well as on the intrinsic aspects that characterize the proposed activity (i.e., the cognitive effort required by exercise) [23]. The most recent scientific evidence showed how the effects of physical activity on cognitive abilities could lead to favorable results in terms of improvements of both low-level cognitive functions related to simple reactions and of higher-level functions such as memory, processing speed, attention, and executive function [24]–[26]. Specifically, many studies argue that, through physical activity, it is possible to contribute to improving the cognitive and instrumental prerequisites necessary to face the first school learning and develop metacognitive skills [27],[28] more easily. This happens because learning is formed through a process of internalization of the activities carried out at the motor and bodily levels. Training on pre-requirements before even starting primary school allows for a better evolution of learning. During the period of kindergarten, the child begins to develop different skills and competences. Although they are not yet scholastically learning, they will be indispensable in Primary School, as they form the basis on which skills will be developed for all future learning. These basic skills are called school pre-requirements and are divided into four macro-categories, within which specific skills can be identified. They include the following: communication and language skills, visual-motor skills, attentional skills, and executive functions (inhibition, working memory, cognitive flexibility, planning, and problem solving). According to an approach of the early identification of learning difficulties and the timely intervention of enhancements, it is desirable to identify valid and effective strategies to develop these skills [29]–[31].

The school environment is an important arena for creating good life habits, initiating changes, and developing cognitive performance. In Italy, there is concern that physical education takes valuable time away from academic work, so much so that it is often the idea that the time allocated for physical education will be drastically reduced by changes in the school curriculum. However, allocating time for physical education does not seem to compromise the academic results. More research is needed to address the benefits of school-based physical activity and to support and motivate relevant groups and policy makers to create policies and environments that support increased physical activity in educational settings [32].

Therefore, the aim of the present study is to explore the relationship between physical activity and the development of school pre-requirements, hypothesizing that the physiological activation of the body and its movement can be an effective means of mediation between the environment and the development of cognitive functions.

## 2. Method

### 2.1. Study design

This study was a randomized controlled trial that was structured to investigate the mediating role function of a school-based physical activity (PA) program on cognitive performance and scholastic prerequisites among school-age children. The research was executed in a kindergarten, where the participants engaged in a PA regimen implemented in a curricular class. Hence, the program of intervention was carried out for a whole school year and encompassed a total of 82 sessions that involved moderate to intense aerobic workouts (MVPA) for the experimental group and standard classes for the control group. The intervention entailed three 60-minute physical activity sessions per week conducted during regular school hours. The assessments were conducted prior to and upon completion of the intervention schemes.

As for the randomization protocol, the simple randomization method through the random number table was used. An electronic tool to generate numerical sequences was used for this purpose. Subsequently, it was established as an allocation rule that subjects that corresponded to “even” digits would fall into the experimental group (EG), while all those that corresponded to “odd” digits would fall into the control group (CG). The research team was kept unaware of the group assignments.

### 2.2. Participants

52 participants, ranging in age from 4 to 5 years (mean age = 4.70, SD = ± 0.46), were selected from a kindergarten situated in the southern region of Italy.

The inclusion criteria was comprised of individuals who possessed the capability to engage in a moderate-to-vigorous intensity aerobic exercise session, those who were enrolled as students in the institution, and those who demonstrated the ability to refrain from any physical activities beyond the confines of the study protocol on the testing days. Children who could not refrain from exercising outside the study guidelines were omitted from the research. It should be noted that participation in the research was entirely voluntary. Parents and their children were educated on the attributes of the research. The researchers guaranteed the confidentiality of the subjects, and the procedure adhered to the guidelines outlined in the Declaration of Helsinki. Written informed consent was obtained from all the parents. The research was carried out between 01 October 2023 and 30 May 2023.

### 2.3. Procedures

Prior to the inaugural school-based physical activity session, a comprehensive briefing was conducted to elucidate the contents of the program, while also confirming the individual motivation of each child. A week prior to the commencement of the intervention, the participants were escorted to the school gymnasium for the purpose of conducting standardized motor assessment tests and a cognitive evaluation. The capability of the participants to engage in a moderate-to-vigorous intensity aerobic exercise session was assessed using the OMNI scale. Moreover, each training session intensity was monitored through an OMNI scale to respect exertion in the MVPA range of a 5 < RPE < 8 and to prevent any differences between training sessions [33].

The participants of the research underwent individual testing, in which each assessment was conducted sequentially, at a consistent time of day, and under comparable conditions. The evaluations were conducted both prior to and following the intervention, which allowed for the examination of the program's effects. The design, supervision, and implementation of all assessments and physical activities were overseen by a pair of physical education instructors, both pre and post the physical activity interventions.

After the administration of the pre-tests, the children of the experimental group participated in an intervention to strengthen the school prerequisites through a recreational-motor path, which lasted 7 months. On the other hand, the children in the control group followed the normal preschool educational program proposed by the curricular teachers, without specific motor content, which mostly included free play either in the classroom or in the garden and preschool reading-writing activities with the use of operational notebooks.

### 2.4. Physical activity intervention

The physical activity intervention was comprised of a warm-up period that lasted 10 minutes, followed by a core session that lasted 40 minutes, and concluded with a cool-down period of 10 minutes. Within the core session, there was a sequence of activities aimed at enhancing engagement, motivation, and pleasure. Evidently, the fitness regimen was formulated to be pleasurable and attractive, with the aim of fostering a comprehensive recognition of one's own capabilities. These components could be succinctly outlined as follows:

- Individual exercises;
- Cooperative activities;
- Exercises and techniques designed to improve lateralization, control, as well as overall and segmental coordination;
- Coordination games and exploratory tasks to acquire proficiency in spatial awareness;
- Utilization of the body parts in activities centered on enhancing focus and mindfulness;
- Physical exercises and leisurely pursuits that involve pairs or teams, often structured to foster unity, mutual support within the group, and confidence in others;
- Utilization of the body to convey, express, and portray both real and imaginary scenarios, personal experiences, emotions, and sentiments; and
- Engagement in rhythmic tasks and games accompanied by music.

## 3. Measures

### 3.1. Motor tests

The assessment examined six subtests that were included in the Motorfit battery [34]. These include the following: 1) Motorfit Locomotor ((Performing forward jumps on one foot (SAP1), executing lateral galloping (GL), and completing forward hopping steps on one foot (SAP2)); and 2) Motorfit Object ((Throwing a ball with one hand (LP), catching a ball with hands (RP), and striking a ball with a tennis racket (CP)).

They were selected based on their straightforward and efficient execution, with minimal equipment being necessary.

### 3.2. Observational Questionnaire for the Early Identification of Learning Disabilities (IPDA)

The Observational Questionnaire for the Early Identification of Learning Disabilities (IPDA) [35] is an observational questionnaire that must be completed by the teacher and is aimed at children in the last year of kindergarten. It allows the early identification of learning difficulties and allows a path that consists of three phases: i) screening; ii) more precise assessment of the state of development of specific skills (administration of IPDA batteries); and iii) targeted rehabilitation intervention for deficit areas.

It consists of 43 items divided into two sections: 1) General skills (items 1–9 behavioral aspects, items 10–11 motor skills, items 12–14 language comprehension, items 15–19 oral expression, items 20–23 metacognition, and items 24–33 other cognitive skills); and 2) Specific skills (items 34–40 pre-literacy, and items 41–43 pre-mathematics). The teacher rates each item following a 4-point Likert scale: 1. Not at all/never; 2. A little/sometimes; 3. Somewhat/most of the time; and 4. A lot/always.

The total score is obtained by summing the scores of the individual items. The total score makes it possible to identify the presence or absence of difficulties in the school prerequisites: high-risk band score ≤ 107; medium-high risk range score between 108 and 118; medium-low risk range score between 119 and 135; and low risk group score ≥ 136.

### 3.3. Statistical Analysis

Statistical analyses were conducted utilizing IBM SPSS, version 25.0 (IBM, Armonk, NY, USA). The data were depicted as group mean (M) values and standard deviations (SD) and were assessed for assumptions of normality, specifically through the Shapiro-Wilk test, and a homogeneity of variances, which involved the Levene test, within the data distributions. An independent sample t-test was executed to assess the group distinctions at baseline, while a two-way analysis of variance (ANOVA) (group (experimental/control) × time (pre/post-intervention)), with repeated measures on the time dimension, was carried out to explore the impact of the intervention on all dependent variables. When the interactions of “Group x Time” attained a statistical significance, subsequent pairwise comparisons were carried out using group-specific post hoc tests, specifically paired t-tests. The extent of the significant “Time x Group” interaction was quantified using partial eta squared (η2p) and was assessed based on the predetermined thresholds: small (η2p < 0.06), medium (0.06 ≤ η2p < 0.14), and large (η2p ≥ 0.14). The effect sizes for the comparisons were evaluated through Cohen's d, with values falling into the following categories: small (0.20 ≤ d < 0.50), moderate (0.50 ≤ d < 0.79), and large (d ≥ 0.80), according to Cohen [36]. The statistical significance level was established at p < 0.05.

## 4. Results

Both groups of participants exhibited no significant variance at the commencement in terms of age, anthropometric traits, and psychological assessments (p > 0.05) (Table 1). The outcomes pre- and post-intervention for all variables of interest are delineated in Table 2.

**Table 1.** Characteristic of participants.

***Variable***	***EG (n = 26)*** ***Mean ± SD***	***CG (n = 26)*** ***Mean ± SD***
Age (y)	4.61 ± 0.49	4.62 ± 0.73
Height (cm)	114.50 ± 1.58	113.84 ± 1.37
Weight (kg)	16.88 ± 3.00	16.90 ± 3.66
Body mass index (kg.m^− 2^ - percentile)	23.20 ± 34.59	24.63 ± 37.28
Sex, n (%)		
Male	17 (65.38)	9 (34.62)
Female	14 (53.85)	12 (46.15)

**Table 2.** Changes after classroom-based PA intervention program.

	**Experimental Group (n = 26)**			**Control Group (n = 26)**		
	**Baseline**	**Post-test**	**Δ**	**Baseline**	**Post-test**	**Δ**
*Motorfit Locomotor*	8.73 (1.15)	9.65 (1.12) †*	0.92 (0.89)	8.68 (1.93)	8.60 (1.87)	-0.08 (0.57)
*Motorfit Object*	8.38 (0.89)	9.61 (1.06) †*	1.23 (0.90)	9.16 (1.31)	8.76 (1.36)	-0.40 (0.64)
*IPDA Questionnaire*	115.57 (1.55)	125.42 (1.55) †*	9.84 (3.10)	115.68 (1.49)	114.64 (2.62)	-1.04 (2.20)

Note: values are presented as mean (± SD); Δ: pre- to post-training changes; †Significant ‘Group x Time' interaction: significant effect of the intervention (*p* < 0.001). *Significantly different from pre-test (*p* < 0.001).

### 4.1. Motor tests

Statistical analysis revealed a significant “Time x Group” interaction for Motorfit Locomotor (F1,_50_ = 23.47, p < 0.001, η2p = 0.31, large effect size*) and Motorfit Object (*F1,_50_ = 56.27, *p* < *0.001*, η2p = *0.53* large effect size). A post-hoc analysis revealed a positive for Motorfit Locomotor *(t = 5.28*, p < 0.001, d = 1.03, large effect size) and Motorfit Object (t = 6.91, *p* < 0.001, *d* = 1.35, large effect size*)* in the *intervention group*. No significant changes were found for the control group (p > 0.05).

### 4.2. IPDA Questionnaire

The statistical analysis revealed a significant “Time x Group” interaction for *IPDA* (*F1*,_50_ *= 214.90*, p *< 0.001*, η2p *= 0.81, large effect size*). A post-hoc analysis revealed a positive decrease in *IPDA* (*t* = 16.15, *p < 0.001*, d *= 3.16, large effect size*) in the intervention group. Additionally, the results indicated that there was a significant main effect of the gender factor (*F1,_50_* (1.22), p *< 0.001*, η^2^ = 0.84], which was higher in the male participants. No significant changes were found for the control group (p > 0.05).

## 5. Discussion

The objective of this study was to explore the correlation between physical activity intervention and scholastic prerequisites among kindergarten children, based on the assumption that the physiological activation due to physical activity plays a crucial role as a mediating factor. The findings from this research indicated that physical activity integrated in the scholastic curriculum proved to be successful in enhancing academic achievement. Conversely, traditional classroom sessions demonstrated a lower efficacy in attaining outcomes that aligned with the objectives outlined in this investigation. In fact, the main significant result that could be observed concerned the improvement in scholastic prerequisites as a consequence of physical activity. This outcome is likely attributed to the phenomenon where children, within the realm of physical activity, can encounter diverse physiological mechanisms that stem from acquiring a new skill. These processes are further bolstered by the intrinsic motivation to effectively participate in the activity on which they were focusing their time and energy [37]–[40]. Moreover, research in the field of neuroscience highlighted a profound interdependence between physical activity and cognitive processes [41]–[43]. According to a conceptualization model proposed by many authors [44]–[48], this interdependence is primarily related to changes that occur in brain function and structure as a consequence of physical activity.

Investigations into the influence of physical activity on cognitive functioning have proposed several mechanisms that could explain this relationship [49]–[53]. Numerous studies published in recent years on this topic have mainly concluded that physical activity is able to determine a series of physiological and structural modifications and adaptations that allow brain cells to create new connections in different cortical areas. In fact, physical activitiy is able to trigger a cascade of neurochemical growth factors capable of changing the entire brain structure. This reflects the brain's ability to adapt to the various cognitive challenges it faces [54],[55].

Stillman, Cohen, Lehman, and Erikson [56] proposed a conceptual model that identified multiple ways in which changes in brain structure, function, and behaviors, as well as in social-emotional functions (mood, motivation, or sleep), could mediate improvements in cognitive performance following physical activity practice. This model was the only one to highlight the idea that the mechanisms which underlie improvements in cognitive performance could be conceptualized on multiple levels: Level 1. Molecular and cellular mechanisms that occur as a result of physical activity; Level 2. Structural and functional modifications and adaptations of the brain; and Level 3. Behavioral and socio-emotional mechanisms. In relation to Level 1, and in particular to molecular mechanisms, exercise seems to exert its positive effects on cognitive functions by modulating the cascades of key growth factors responsible for synaptic plasticity. The concept of *plasticity* is fundamental to understand how exercise can optimize brain function by promoting the quality of learning [57]. Neuroplasticity is a constant and continuous process capable of modifying existing neuronal networks by mediating the structural and functional adaptations of synapses in response to changes in behavior [58]; additionally, this is in relation to the exercise and repeatability of similar networks. Neuroplasticity is a sort of structural and functional disposition of our nervous system to change as a result of stimuli from the environment: the richer and more varied the stresses, the more our brain will be able to adapt and modulate itself in response to them [59]. In fact, the human brain is not made up of fixed and immutable neural circuits; however, the cerebral synaptic network and the structures connected to it are capable of actively reorganizing themselves thanks to practice and experience [60] by reprogramming their neuronal networks. One of the molecules believed to be involved in these processes is the Brain-Derived Neurotrophic Factor (BDNF), whose levels are increased following physical activity and closely correlate with the improvement in learning and memory described in rodents that underwent physical training [61]. BDNF is a polypeptide known as one of the major modulators of brain plasticity [62]. By increasing BDNF synthesis, physical activity would induce the creation of an ideal neurobiological environment that would become a significantly predisposed ground to accommodate the changes and adaptations induced by learning processes [63]. The cognitive aspects that would seem to benefit most from this increase in the BDNF protein are memory and executive functions, as well as an improved performance related to attention, inhibitory control, work speed, and visual learning [64]. Two other molecules potentially involved in the effects of physical activity on cognitive functions are Vascular Endothelial Growth Factor (VEGF) and Insulin-Like Growth Factor 1 (IGF1), which are responsible for activating neoangiogenesis processes in the brain [65]. VEGF, which is a subfamily of growth factors involved in vasculogenesis and angiogenesis, promotes the proliferation of neuronal precursors and provides a vascular heritage that is useful for the growth of neurons. IGF-1, which is a protein hormone with a molecular structure similar to insulin, is important for the development and maintenance of the nervous system. Physical activity can increase gene expression and protein levels of BDNF, IGF-1, and VEGF in different regions of the brain, particularly in the hippocampus [66].

As far as cellular mechanisms are concerned, they are associated with phenomena related to neurogenesis and angiogenesis as neural mechanisms that mediate the beneficial cognitive effects of physical activity [67]. They are the result of increased oxygenation and irrigation of tissues, as well as an increased metabolic activity in response to exercise [68]. Angiogenesis, namely the development of new blood vessels, and neurogenesis (i.e., the development of new neurons), are complex cellular changes that result from the increased production of growth factors and upregulated molecular cascades [69]. The process of neurogenesis mainly occurs in the hippocampus (especially in the dentate gyrus), which is an area specialized in spatial learning and the consolidation of short- and long-term memory. Neurogenesis phenomena, starting from neonatal cells, could help to explain the effects of physical activity on learning and memory functions [70].

Most of the studies that have examined the mechanisms which underlie the level 2 analysis have focused on the effects of physical activity in relation to brain structure, particularly in regard to gray matter volume and changes in the white matter microstructure, especially in the temporal and prefrontal region. These modifications would partly explain the cognitive improvements after exercise [71],[72].

Chaddock et al. [73] systematically demonstrated the effects of physical activity on brain structure and function and the existence of a positive relationship between aerobic fitness, brain volume, and cognitive abilities. In fact, the authors argue that higher levels of aerobic fitness were related to higher academic achievement, improved cognitive abilities, larger brain structures, and higher brain function in school-age children (9–10 years). Through the use of functional magnetic resonance imaging (fMRI), the authors found that fitter children have a high ability to activate the frontal and parietal brain regions important for monitoring, maintaining, and strategizing higher-level cognitive control skills, which are skills that are important to achieve an improved learning performance. Additionally, they showed that fitter children had larger bilateral hippocampal volumes and higher performances on memory tasks compared to less fit children. Interestingly, these results differed from the results of the studies used in the meta-analysis by Sibley and Etnier [17], in which the authors suggested that physical activity was not related to memory skills in children aged 4 to 18 years [74].

Hillman et al. [75] showed that children that participated in at least 70 minutes per day of intense physical activity for nine months, directed toward increasing aerobic capacity, significantly improved the brain function and behavioral indices of executive control. The authors suggested that fitness-related benefits appeared to follow an observed dose-response relationship between the participation rate, brain function, and executive control, and showed that brain and behavioral changes were a function of the degree of participation in the physical activity program. Hillman and colleagues found that fitter children outperformed less fit children in Eriksen's Flanker test (a conflict resolution test). In addition to the behavioral measures, the authors studied event-related brain potentials (ERPs) derived from electroencephalographic (EEG) activity through 64 electrodes that collected P3 amplitude and latency measurements during cognitive tasks. They showed that, compared to controls, children that participated in the physical activity program showed more pronounced changes in neural indices of attention (P3 amplitude), processing speed (P3 latency), and an improved performance during executive control tasks, which reflected the greater allocation of attentional resources and a greater cognitive processing speed.

The data obtained in this investigation also suggested that statistically notable gender disparities were observed between boys and girls. Indeed, scores acquired by boys were elevated; these findings corroborate certain research [76]–[80], which suggested that boys universally achieved higher scores. These global disparities concerning gender could be attributed, in part, to the elevated scores acquired by girls in manual dexterity [81] and in balance [82], which were possibly generated by the cultivation of practices of stereotypical activities, and various sports activities, among boys and girls. Young elaborated on the distinctions in human throwing and hitting conduct from an evolutionary standpoint. Early humans sustained themselves by throwing rocks and wielding clubs. Women allocated more resources towards reproduction, while men were more inclined towards being hunters and fighters. These sorts of trends were passed down through natural selection [83]. A prior examination theorized that throwing is more likely an inherent ability whose progression is biologically predetermined and rather challenging to be impacted by nurture; the same might hold true for striking [84]. Moreover, societal aspects and behavioral tendencies might contribute to gender disparities in skillfulness in object manipulation.

This study offered support for the positive correlation between physical activity and academic performance; however, certain limitations within the research necessitate further examination. In particular, the study was constrained by the fact that it only included students from a single educational institution, which limited the generalizability of the results to students from diverse school settings or varying demographic backgrounds. Another limitation concerned the fact that mid-term assessments were not conducted over 7 months. Furthermore, the small sample size of 52 participants due to the difficulties in engaging participants served as another notable limitation. Lastly, the study also failed to assess the socio-emotional variables associated with physical activity and the school environment, which presented another area of limitation. The scientific evidence described above implies a series of physiological, molecular, cellular, and cerebral processes involved in the relationship between physical activity and cognition; however, at a more in-depth level of analysis, it is likely that it also exerts its positive influence in relation to human and socio-emotional behavior [53]. Moreover, the study fails to investigate a broad spectrum of age groups and was limited to data gathered within a specific timeframe. Hence, it is advisable that future studies should examine comparable variables across a more diverse and extensive sample. Nonetheless, the results obtained could offer valuable insights for upcoming research endeavors. Thus, the effectiveness of this study was bolstered by a strategic approach that enhances not only physical fitness and academic prerequisites, but also scholastic performance.

## 6. Conclusions

The findings derived from the current study enable us to assert with increased certainty that engaging in physical activity leads to significant effects that facilitate enhancements in cognitive function, thereby offering various avenues to enhance the educational methods of school-aged individuals. Moreover, facilitating regular physical activity among children during the academic day has the potential to enhance academic performance and simultaneously foster a balanced mind-body growth. Hence, given the acknowledged positive correlation between physical activity and academic requirements, physical activity represents a beneficial avenue to encourage a dynamic lifestyle among school-aged children. Since the educational institution serves as the primary environment where children spend most of their time, it is imperative for the school setting to facilitate opportunities to meet the recommended levels of physical activity through more robust initiatives to extend the duration dedicated to physical exercise.
